# Potential of Artificial Intelligence in Addressing Japan’s Clinical Research Disconnection

**DOI:** 10.31662/jmaj.2024-0084

**Published:** 2025-06-13

**Authors:** Yudai Kaneda, Akihiko Ozaki

**Affiliations:** 1 School of Medicine, Hokkaido University, Hokkaido, Japan; 2 Department of Breast and Thyroid Surgery, Jyoban Hospital of Tokiwa Foundation, Fukushima, Japan

**Keywords:** artificial intelligence (AI), evidence-based medicine (EBM), Japanese University Hospitals, medical ethics, patient care

## Abstract

In February 2023, a report from the Japanese Ministry of Education, Culture, Sports, Science and Technology highlighted that half of the assistant professors at university hospitals spend five hours or less per week on research, with 15% not engaging in research at all. This disconnect between clinical practice and research may be exacerbated by work-style reforms predicting difficulties in allocating time for student guidance and dedicated research at Japanese university hospitals. This study proposes the integration of artificial intelligence (AI) tools, such as ChatGPT, to streamline administrative duties and enhance research productivity, suggesting a need for immediate development of ethical guidelines for AI use in healthcare to improve efficiency and patient care quality.

In February 2023, the Japanese Ministry of Education, Culture, Sports, Science and Technology provided data that half of assistant professors at university hospitals dedicate only five hours or less per week to research, and alarmingly, 15% do not engage in research at all ^(1)^, which raises significant concerns for the medical community in Japan. Moreover, with the initiation of the work-style reform, approximately 80% of university hospitals anticipate hindrances in setting aside time for one-on-one student guidance and dedicated research ^(1)^. This data underscores a disconnect between clinical practice and rigorous research in Japan’s university hospitals.

Medical practice hinges on the professional responsibility of physicians to provide the best possible care for their patients while continuously advancing medical knowledge ^(2)^. Furthermore, physicians are expected to practice evidence-based medicine, necessitating that they stay updated on the latest research findings and incorporate them into their daily practice ^(3)^. In this context, the importance of physicians engaging in medical research is emphasized ^(4)^, whereas the current environment in Japan is characterized by a distinct chasm between clinical practice and research, threatening the foundational principles of medical professionalism and the ability to deliver optimal, evidence-based care.

Moreover, a closer examination of doctors’ daily routines reveals a concerning trend. An observational study conducted at a Japanese university hospital found that physicians spent more time on indirect patient care (approximately 3.6 hours) than on direct patient interactions (approximately 2.9 hours), underscoring a significant portion of the workday dedicated to indirect care activities ^(5)^. Similarly, research in the United States reports that direct patient interaction comprises less than a third of a physician’s workday, with nearly half devoted to electronic record-keeping and other administrative tasks ^(6)^. This disproportionate allocation of time away from direct patient care, a problem not confined to Japan but prevalent in healthcare settings worldwide, highlights the need for efficient technologic solutions.

Although there was an observed increase in the understanding of professional behaviors among Japanese medical residents from 2005 to 2013 ^(7)^, the current situation suggests that these improvements are not adequate to meet the evolving demands of modern medical practice, especially in the unique environment of university hospitals. These institutions, which blend medical practice with educational responsibilities, necessitate a novel approach to physician development.

In this context, given the ability of artificial intelligence (AI) to process vast volumes of data swiftly, there is a tangible opportunity to reduce the time physicians spend on administrative tasks. For instance, our prior research revealed that using a free recording application in conjunction with ChatGPT, medical records summaries of a quality comparable to those created by veteran internists in the Subjective, Objective, Assessment, and Plan format could be instantaneously generated ^(8)^. These pioneering efforts would pave the way for enhanced patient interactions and open avenues for dedicated research hours, thereby addressing the concerns.

In conclusion, integrating AI tools such as ChatGPT into physicians’ workflows worldwide can revolutionize task efficiency, research, and patient communication, significantly enhancing healthcare quality. However, although it has been a year since the first release of ChatGPT in November 2022, and its practical application has been advancing in other departments such as administrative circles, there has been insufficient discussion regarding AI utilization in medical settings. Moreover, maintaining high barriers to application will further delay the experimentation and refinement needed for ideal implementation. Therefore, it is imperative to recognize that AI has both advantages and drawbacks and to commence the development of clear ethical regulations and guidelines as soon as possible, instead of polarizing the discussion into whether incorporating AI into healthcare is beneficial or harmful.

## Article Information

### Conflicts of Interest

Dr. Ozaki received personal fees from MNES, Kyowa Kirin Inc., Becton, Dickinson and Company, Pfizer, Daiichi Sankyo Inc and Taiho Pharmaceutical Co., Ltd., outside the scope of the submitted work.

### Author Contributions

Yudai Kaneda conceived and designed the study and wrote the article. Akihiko Ozaki critically revised the article. Both authors read the final draft and approved submission.
